# Determinants of trends in neonatal, post-neonatal, infant, child and under-five mortalities in Tanzania from 2004 to 2016

**DOI:** 10.1186/s12889-019-7547-x

**Published:** 2019-09-09

**Authors:** Felix Akpojene Ogbo, Osita Kingsley Ezeh, Akorede O. Awosemo, Ifegwu K. Ifegwu, Lawrence Tan, Emmanuel Jessa, Deborah Charwe, Kingsley Emwinyore Agho

**Affiliations:** 1 Translational Health Research Institute (THRI), School of Medicine, Western Sydney University, Penrith, NSW 2751 Australia; 2 General Practice Unit, Prescot Specialist Medical Centre, Welfare Quarters, Makurdi, Benue State Nigeria; 3 School of Science and Health, Western Sydney University, Campbelltown Campus, Locked Bag 1797, Penrith, NSW 2571 Australia; 4 Department of General Practice, School of Medicine, Western Sydney University, Penrith, NSW 2751 Australia; 5 Tanzania Food and Nutrition Centre, No 22. Ocean Road, Dar es Salaam, Tanzania

**Keywords:** Neonatal, Infant, Child, Under-five, Mortality, Tanzania

## Abstract

**Background:**

Under-five mortality is still a major health issue in many developing countries like Tanzania. To achieve the Sustainable Development Goal target of ending preventable child deaths in Tanzania, a detailed understanding of the risk factors for under-five deaths is essential to guide targeted interventions. This study aimed to investigate trends and determinants of neonatal, post-neonatal, infant, child and under-five mortalities in Tanzania from 2004 to 2016.

**Methods:**

The study used combined data from the 2004–2005, 2010 and 2015–2016 Tanzania Demographic and Health Surveys, with a sample of 25,951 singletons live births and 1585 under-five deaths. We calculated age-specific mortality rates, followed by an assessment of trends and determinants (community, socioeconomic, individual and health service) of neonatal, postneonatal, infant, child and under-five mortalities in Cox regression models. The models adjusted for potential confounders, clustering and sampling weights.

**Results:**

Between 2004 and 2016, we found that neonatal mortality rate remained unchanged, while postneonatal mortality and child mortality rates have halved in Tanzania. Infant mortality and under-five mortality rates have also declined. Mothers who gave births through caesarean section, younger mothers (< 20 years), mothers who perceived their babies to be small or very small and those with fourth or higher birth rank and a short preceding birth interval (≤2 years) reported higher risk of neonatal, postneonatal and infant mortalities.

**Conclusion:**

Our study suggests that there was increased survival of children under-5 years in Tanzania driven by significant improvements in postneonatal, infant and child survival rates. However, there remains unfinished work in ending preventable child deaths in Tanzania.

**Electronic supplementary material:**

The online version of this article (10.1186/s12889-019-7547-x) contains supplementary material, which is available to authorized users.

## Background

In the past four decades, considerable reductions in under-five mortality have occurred globally, with significant improvements in child survival in many countries. A recent study estimated that under-five mortality rate (U5MR, defined as the probability of dying before the fifth birthday [1]) had declined globally from 69 deaths in 2000 to 38 deaths per 1000 live births in 2016 – a drop of approximately 45% [2]. These improvements have been attributed to micro- and macro-economic growth; improved female education; lower fertility rates; strengthened global, regional and national public health programmes. In addition, the Millennium Development Goal (MDG) agenda and increased developmental assistance for health also played significant roles in reducing global and national U5MR [3].

Despite this progress, differences in U5MR between low-income and high-income countries remain huge (76 deaths and 7 deaths per 1000 live births in low-income and high-income countries, respectively) [2]. In 2016, over 70% of the burden of global U5M occurred in sub-Saharan African and South Asian countries [2]. For sub-Saharan Africa, the burden of U5M has been uneven, with Nigeria, Democratic Republic of Congo and Ethiopia accounting for over 60% of U5M in the region [2]. Most importantly, there was huge heterogeneity in absolute levels of U5M and neonatal mortality during the MDG era in Africa [4]. In the post-MDG period, the global community has agreed on a new set of targets, the Sustainable Development Goals (SDGs), with SDG-3.2 aiming to reduce under-five mortality to at least as low as 25 per 1000 live births by the year 2030 [5]. Although the SDG agenda focuses on both developed and developing countries, SDG-3.2 is particularly relevant for developing countries given the current estimate of U5M in developed countries [2].

In Tanzania, between 1999 and 2015, infant mortality rate (IMR, defined as the probability of dying before the 1st birthday [1]) had declined by approximately 57% and U5MR fell by 54% [6], with a recent estimate ranging between 48 [6] and 60 [7] per 1000 live births. This progress has been attributed to strong political will, improved health care systems and programmes, and improvements in income per capital [8, 9]. However, additional efforts are needed, especially in the achievement of SDG-3.2. For further improvement in U5M, nationally representative household studies that focused on trends and determinants of deaths for children younger than 5 years are needed for effective prioritising of scarce maternal and child health resources in Tanzania [10].

Previous studies from rural Tanzania indicated that poor maternal education, limited exclusive breastfeeding, low socioeconomic status and indoor air pollution were risk factors for child mortality [11, 12]. Additionally, a population-based study conducted in the pre-MDG period indicated that short birth interval, teenage pregnancy and history of child death increased the risk of infant and child mortalities in Tanzania [12].

In Tanzania, maternal, newborn and child health programmes are being implemented in the post-MDG era to ensure sustained improvement in child survival and longevity of life [8, 10]. Nationally representative and country-specific evidence on the risk factors for neonatal, post-neonatal, infant, child and U5 mortalities is essential to further improve U5MRs, particularly due to the dearth of vital registration or population-based surveillance data at the national level in Tanzania [13]. Similarly, the full implementation of the SDG-3 agenda will also require locally-relevant data to guide efforts and/or refinement of programmes to high-priority groups. The present study aimed to investigate the trends and determinants of neonatal, post-neonatal, infant, child and U5 mortalities in Tanzania between 2004 and 2016.

## Methods

### Data sources

The study used combined Tanzania demographic and health survey (TDHS) data sets (that is, the children datasets for the years 2004–2005, 2010 and 2015–2016) to estimate trends and also to increase the sample size and statistical power, consistent with previous studies [14–17]. The surveys were conducted by the National Bureau of Statistics (NBS), Dar es Salaam, Tanzania and Inner City Fund (ICF) International, Maryland, USA. Using standardised questionnaires, the surveys collected maternal, child and household data (including births and deaths of children under-5 years) from women aged 15–49 years who participated in the surveys, with an average response rate of 98%. A total weighted sample of 26,953 live births, consisting of singleton and multiple births occurred during the study period (2004–2016).

We excluded 1002 multiple births from the total sample because multiple births have a greater mortality risk from preterm birth and pregnancy complications compared to singletons [18]. The present study used a total weighted sample of 25,951 singleton live births (2004–2005 = 8394; 2010 = 7939 and 2015–2016 = 9618). The data were weighted to account for unequal selection probabilities in each of the clusters used for the surveys and ensure the representativeness of the survey results at the national level [6]. Additionally, the analyses were restricted to births within 5-years before the surveys to reduce the potential effect of recall bias given differences in the delivery time-points and the period of the interviews [14]. Additional information on the survey methodology (such as sampling procedure and questionnaire details) has been described elsewhere [6].

### Potential risk factors

The study factors were selected based on the conceptual model of determinants of child survival in developing countries as described by Mosley and Chen [19] and previously published studies [14, 20]. The study factors were classified into four main groups (community, socioeconomic, individual and health service factors). Community-level factors included place of residence, while socioeconomic level factors included household wealth index, maternal and paternal education and maternal employment status. The household wealth index is a relative measures of the economic status of households interviewed during the surveys. The wealth index was calculated as a score of household assets (i.e., television, radio, refrigerator, car, bicycle, motorcycle, source of drinking water, type of toilet facility, electricity and type of building materials used in the place of dwelling), using a principal component analysis [21]. In the TDHS, the household wealth index was categorised into five quintiles: poorest, poorer, middle, richer and richest. In the present analysis, however, the household wealth index was re-categorised into three groups with the bottom 40% of households was arbitrarily classified as ‘poor’, the next 40% as ‘middle’ households and the top 20% as ‘rich’ households [22].

In this study, we excluded antenatal and postnatal care data as the study was based on all singleton births within the 5-year period preceding the surveys. In the 2015–2016 TDHS, antenatal and postnatal care information was only recorded for the last birth (or most recent birth) of the mother interviewed in the 5 years prior to the survey to reduce the potential effect of recall bias that may arise from antenatal and postnatal care visits and/or services use. In addition, birth weight of newborns at birth was not included because nearly 50% of newborns were not weighed at the time of birth. However, perceived baby size by mothers was used in place of baby weight at birth [14]. Individual-level factors comprised maternal and child characteristics. Maternal characteristics incorporated in the study were maternal age and body mass index, birth order, birth interval and desire for pregnancy; and child characteristics included perceived baby size by mothers and gender. Health service level factors consisted of birthplace, mode of delivery and type of delivery assistance.

### Outcome

The study dependent variables were age-sepcific mortality rates which included neonatal mortality (defined as the probability of dying within the 1st month of life); post-neonatal mortality (defined as the difference between infant and neonatal mortality), infant mortality (measured as the probability of dying before the 1st birthday), child mortality (assessed as the probability of dying between the 1st and 5th birthdays) and total under-5 mortality (defined as the probability of dying before the fifth birthday) [1]. A death of an infant within the specified periods was coded as ‘1’ and no death coded as ‘0’. All mortality rates were expressed in 1000 live births. IMR is a composite indicator of neonatal mortality rate (NMR) and post-neonatal mortality rate, while U5MR is a composite measure of IMR and child mortality rate (CMR). The selection of these age categories was based on the reporting of sub-categorisation of U5M in the DHS [6] and previous studies to inform targeted interventions [7, 14, 20, 23].

### Statistical analysis

Mortality rates (and corresponding 95% confidence intervals, CI) by year of the survey and the study variables were estimated using ‘syncmrates’ command in Stata as described by Rutstein and Rojas [24]. This step was followed by univariate and multivariable analysis using Cox proportional hazard regression models. Cox regression model was used for modelling the time to a specified event (neonatal, post-neonatal, infant, child or under-5 death). We estimated time to event (death) in days within the specified age range and time period based on varied information on age at death recorded by TDHS, for consistency across all the study outcomes [25]. Crude hazard ratios (HRs) for factors associated with the study outcomes were estimated in models. Adjusting for potential confounding variables, a multivariable analysis that independently examined the impact of each factor on each of the study outcomes was conducted. We used the ‘svy’ command to adjust for cluster variations and weights in analyses and ‘tobit’ and ‘truncreg’ commands to determine the number of censoring and truncation in Stata software version 13.0 (Stata Corp, College Station, Texas, USA).

Multivariable Cox regression analysis was performed using a stage model approach, consistent with previous studies [20, 26]. This approach permits more distal factors to be appropriately assessed without interference from individual and healthcare service level factors. Each level factor was entered into the model one at a time to assess their relationship with the study outcomes. In all, a four-stage model approach was conducted. At the start, community-level factors and the outcome variables for each year of the survey were entered into the baseline model. A manually processed stepwise backward elimination was performed to identify variables significantly associated with the study outcomes at 0.05 significance level, which were retained *(stage 1 model).* In the second stage, all socioeconomic variables were investigated with those variables that were significantly associated with the mortality outcomes in *stage 1 model,* and again variables with *p*-value < 0.05 were retained *(stage 2 model)*. In the third stage, individual-level factors (maternal and child characteristics) were assessed with those variables that were significantly related to the mortality outcomes in *stage 2 model*. As before, those variables that satisfied the 5% significance level were retained *(stage 3 model).* In the final stage, a similar procedure was adopted for the health service variables, which were investigated with those variables significantly associated with mortality outcomes in *stage 3 model*. For each of the significant variables in the final model, the HRs and their 95% confidence intervals (CIs) were used to measure the magnitude of effect of factors associated with the various outcomes. Results of the full analytical strategy for each of the study outcomes are presented in Additional file 1 (Table S1–S5).

### Ethics

The DHS project obtained the ethical approvals from the National Health Research Ethical Committee in Tanzania before the surveys were conducted. Participants provided written informed consents prior to being allowed in the surveys. Data used in this study were anonymous, and Measure DHS/ ICF International approved the use of the data for this study.

## Results

### Counts of neonatal, post-neonatal, infant, child and under-five mortalities by study factors

Of the total weighted sample of 25,951 singleton live births of children under-five, there were 618 neonatal deaths, 563 post-neonatal deaths, 1182 infant deaths, 403 child deaths and 1585 under-five deaths from 2004 to 2016 in Tanzania (Additional file 1: Table S6). NMR was higher in urban areas compared to rural areas, while PMR was higher in rural areas compared to urban areas. There were variations in the morality rates across the study factors (Table 1).

**Table 1:** Distribution of all childhood mortality rates with 95% confidence interval (CI), TDHS 2004–2016

Variables	NMR	PMR	IMR	CMR	U5MR
*Community level factor*					
Residence type					
Urban	33 (29 ― 38)	18 (14 ― 21)	51 (45 ― 57)	17 (13 ―20)	68 (61 ― 74)
Rural	21 (19 ― 23)	23 (21 ― 25)	44 (41 ― 47)	15 (14 ―17)	59 (56 ― 63)
*Socioeconomic level factor*					
Household wealth index					
Rich	31 (25 ― 36)	19 (15 ― 23)	50 (43 ― 57)	10 (7 ― 13)	60 (53 ― 68)
Middle	24 (21 ― 27)	21 (18 ― 24)	45 (41 ― 49)	17 (14 ―19)	62 (57 ― 67)
Poor	21 (19 ― 24)	23 (20 ― 26)	44 (40 ― 48)	16 (14 ―19)	61 (56 ― 65)
Mother’s education					
Secondary or higher	28 (21 ― 35)	12 (7 ― 16)	41 (32 ― 49)	5 (2 ― 8)	45 (36 ― 54)
Primary	25 (23 ― 28)	22 (19 ― 24)	47 (44 ― 50)	16 (14 ―18)	63 (59 ― 67)
No education	18 (15 ― 22)	26 (22 ― 30)	44 (39 ― 49)	18 (15 ―22)	62 (56 ― 68)
Mother’s working status					
Working	23 (21 ― 25)	23 (21 ― 26)	46 (43 ― 49)	16 (14 ―18)	62 (59 ― 66)
Not working	28 (23 ― 33)	15 (11 ― 19)	43 (37 ― 49)	13 (10 ―17)	56 (49 ― 63)
Father’s education					
Secondary or higher	31 (24 ― 38)	15 (10 ― 19)	46 (38 ― 54)	8 (4 ― 11)	53 (45 ― 63)
Primary	22 (20 ― 25)	21 (19 ― 23)	44 (40 ― 47)	18 (16 ―20)	61 (57 ― 65)
No education	20 (15 ― 24)	29 (24 ― 34)	49 (42 ― 55)	14 (10 ―17)	62 (54 ― 70)
*Individual level factor*					
Mother’s age (Years)					
30―39	24 (21 ― 27)	20 (17 ― 23)	44 (40 ― 48)	15 (13 ―18)	59 (54 ― 64)
< 20	37 (28 ― 47)	18 (11 ― 24)	55 (43 ― 66)	11 (6 ― 16)	66 (53 ― 78)
20―29	23 (20 ― 25)	23 (20 ― 25)	45 (42 ― 49)	16 (14 ―18)	61 (57 ― 65)
40―49	21 (15 ― 27)	26 (19 ― 32)	47 (38 ― 56)	18 (13 ―24)	65 (54 ― 75)
Mother’s body mass index (MBMI, kg/m2)					
MBMI > 18.5	24 (22 ― 26)	22 (20 ― 24)	46 (43 ― 48)	16 (14 ―17)	61 (58 ― 64)
MBMI ≤18,5	23 (16 ― 30)	19 (13 ― 25)	43 (33 ― 52)	16 (11 ―22)	58 (48 ― 69)
Wanted pregnancy at the time					
Wanted then	24 (22 ― 27)	23 (21 ― 26)	48 (45 ― 51)	16 (14 ―18)	64 (60 ― 67)
Wanted later	20 (17 ― 24)	13 (10 ― 16)	34 (29 ― 38)	15 (12 ―18)	48 (42 ― 54)
No more	24 (15 ― 34)	22 (13 ― 30)	46 (34 ― 59)	9 (3 ― 15)	55 (41 ― 69)
Birth order					
Second or third child	36 (30 ― 41)	24 (19 ― 30)	60 (52 ― 68)	33 (24 ― 41)	91 (81 ― 101)
Fourth or more	20 (16 ― 24)	24 (20 ― 28)	44 (39 ― 49)	33 (26 ― 40)	75 (68 ― 83)
Birth interval					
Interval ≤ 24 months	30 (23 ― 37)	31 (24 ― 38)	61 (50 ― 71)	40 (28 ― 51)	98 (82 ― 114)
Interval > 24 months	18 (15 ― 21)	23 (20 ― 26)	41 (38 ― 45)	33 (27 ― 39)	73 (66 ― 80)
Birth order and birth interval					
2nd or 3rd child, interval > 2	18 (15 ― 22)	20 (16 ― 23)	38 (34 ― 43)	15 (12 ―18)	53 (48 ― 59)
First child	35 (31 ― 40)	21 (18 ― 25)	57 (51 ― 63)	15 (12 ―18)	72 (65 ― 79)
2nd or 3rd child, interval ≤ 2	26 (19 ― 34)	25 (18 ― 33)	52 (42 ― 62)	17 (12 ―24)	69 (58 ― 81)
4th or more child, interval > 2	18 (15 ― 21)	21 (18 ― 24)	39 (34 ― 43)	15 (13 ―18)	54 (49 ― 59)
4th or more child, interval ≤ 2	32 (25 ― 39)	29 (22 ― 36)	61 (51 ― 71)	17 (12 ―23)	78 (67 ― 89)
Sex					
Female	18 (16 ― 21)	22 (20 ― 25)	41 (37 ― 44)	14 (12 ―17)	55 (51 ― 59)
Male	29 (26 ― 32)	21 (19 ― 24)	50 (46 ― 54)	17 (14 ―19)	67 (62 ― 71)
Mother’s perceived baby size at birth					
Average or larger	20 (18 ― 22)	21 (19 ― 23)	41 (38 ― 43)	15 (14 ―17)	56 (53 ― 59)
Small or very small	57 (47 ― 67)	26 (20 ― 33)	83 (72 ― 95)	20 (15 ―26)	104(90―117)
*Health service factor*					
Delivery assistance					
Health professional	27 (23 ― 31)	23 (19 ― 26)	49 (45 ― 54)	16 (14 ―19)	66 (60 ― 72)
Non-health professional	22 (19 ― 24)	21 (19 ― 23)	43 (39 ― 46)	15 (13 ―17)	57 (53 ― 62)
Mode of delivery					
Non-caesarean	23 (21 ― 25)	21 (20 ― 23)	44 (42 ― 47)	16 (14 ―18)	60 (57 ― 63)
Caesarean section	48 (35 ― 61)	24 (15 ― 33)	71 (55 ― 86)	6 (2 ― 11)	78 (62 ― 94)
Place of delivery					
Health facility	28 (24 ― 33)	18 (15 ― 22)	47 (41 ― 52)	14 (11 ―17)	61 (54 ― 67)
Home	20 (17 ― 22)	23 (20 ― 26)	42 (39 ― 46)	17 (15 ―20)	60 (55 ― 64)

### Trends in neonatal, post-neonatal, infant, child and under-five mortalities

**Fig. 1:**
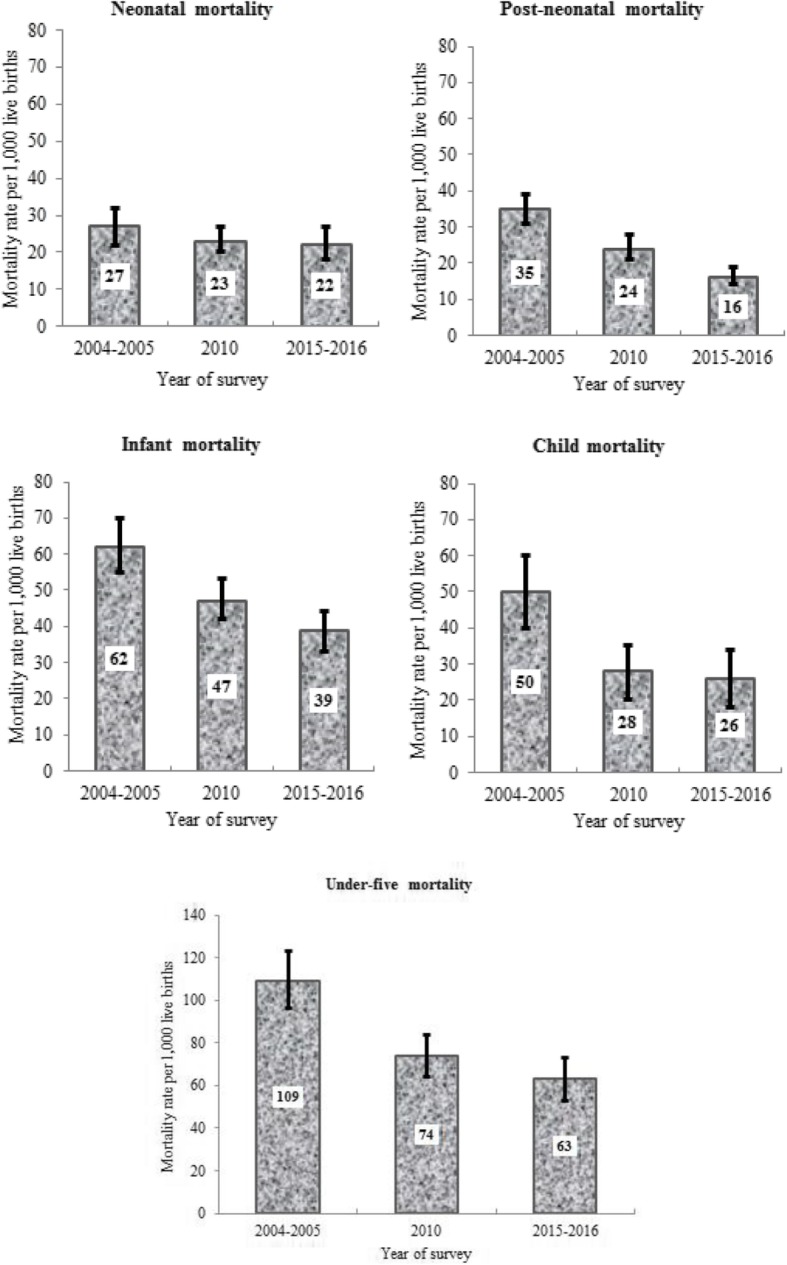
Neonatal, postneonatal, infant, child and under-five deaths per 1000 live births (singleton), with 95% confidence intervals in Tanzania, 2004–2016

Between 2004 and 2016, the analysis showed that NMR has remained relatively unchanged, while PMR has halved, a decrease of 54%, from 35 deaths per 1000 live births in 2004 to 16 deaths per 1000 live births in 2016. IMR decreased by approximately 37%, from 62 deaths to 39 deaths per 1000 live births; CMR fell by 48%, from 50 deaths to 26 deaths per 1000 live births, and U5MR declined by 42%, from 109 deaths to 63 deaths per 1000 live births over the study period [Fig. 1].

### Risk factors for neonatal mortality (0–28 days)

Neonates born to mothers who resided in rural areas had a significantly lower risk of death compared to those whose mothers resided in urban areas (Table 2). In comparisons to mothers aged 30–39 years, neonates of mothers aged less than 20 years had a higher risk of death. The analysis revealed that male neonates were more likely to die compared to their female counterparts, and neonates delivered by caesarean section had a higher likelihood of death compared to those who were delivered by vaginal delivery. Neonates with small or very small perceived baby size were more likely to die compared to those perceived as average or larger-sized. Fourth or higher birth order with a short birth interval ≤ 2 years was also a risk factor for neonatal mortality (Table 2).

**Table 2:** Factors associated with neonatal, post-neonatal, infant, child and under-five mortalities in Tanzania, 2004–2016

	Neonatal (0–28 days)	Post-neonatal (1–11 months)	Infant (0–11 months)	Child (12–59 months)	Under-five (0–59 months)
Variables	AHR (95%CI)	AHR (95%CI)	AHR (95%CI)	AHR (95%CI)	AHR (95%CI)
Year of survey					
2004–2005	*Ref*	*Ref*	*Ref*	*Ref*	*Ref*
2010	0.79 (0.61–1.02)	0.65 (0.51–0.84)	0.71 (0.59–0.85)	0.57 (0.43–0.76)	0.69 (0.59–0.80)
2015–2016	0.75 (0.59–0.95)	0.47 (0.36–0.61)	0.62 (0.52―0.74)	0.50 (0.37–0.68)	0.60 (0.51–0.70)
Residence type					
Urban	*Ref*		*Ref*	*Ref*	*Ref*
Rural	0.61 (0.48–0.78)		0.80 (0.66―0.96)	0.55 (0.38–0.81)	0.79 (0.67–0.93)
*Socio-demographic factor*					
Household wealth index					
Rich	–	–	–	*Ref*	–
Middle	–	–	–	2.03 (1.19–3.46)	–
Poor	–	–	–	2.18 (1.25–3.81)	–
Mother’s education					
Secondary or higher	–	–	–	*Ref*	*Ref*
Primary	–	–	–	2.47 (1.21–5.07)	1.38 (1.06–1.80)
No education	–	–	–	2.68 (1.27–5.67)	1.32 (0.99–1.77)
Mother’s age (Years)					
30―39	*Ref*	*Ref*	*Ref*	–	–
< 20	3.30 (2.07–5.26)	6.50 (3.39–12.5)	4.50 (3.03–6.68)	–	–
20―29	0.97 (0.72–1.32)	1.93 (1.39–2.68)	1.34 (1.05–1.71)		
40―49	0.76 (0.53–1.09)	0.98 (0.65–1.49)	0.88 (0.66–1.16)		
Wanted pregnancy at the time					
Wanted then	–	*Ref*	–	–	*Ref*
Wanted later	–	0.71 (0.51–0.99)	–	–	0.78 (0.66–0.91)
Unwanted	–	1.32 (0.71–2.49)	–	–	0.89 (0.63–1.26)
Father’s education^+^					
Secondary or higher	–	*Ref*	–	–	–
Primary	–	1.13 (0.77–1.65)	–	–	–
No education	–	1.53 (1.02–2.29)	–	–	–
Birth rank and birth interval^+^					
2 or 3 child, interval > 2	*Ref*	*Ref*	*Ref*		*Ref*
First child	1.45 (1.09–1.92)	0.82 (0.59–1.15)	1.10 (0.89―1.35)		1.39 (1.18–1.63)
2 or 3 child, interval ≤ 2	1.42 (0.97–2.08)	1.19 (0.78–1.80)	1.27 (0.98―1.64)		1.43 (1.14–1.79)
4 or more child, interval > 2	1.08 (0.77–1.53)	1.64 (1.14–2.36)	1.28 (0.98―1.67)		1.04 (0.88–1.24)
4 or more child, interval ≤ 2	1.91 (1.30–2.79)	2.03 (1.33–3.12)	1.84 (1.37―2.47)		1.58 (1.27–1.98)
Child sex					
Female	*Ref*	–	*Ref*	*–*	*Ref*
Male	1.59 (1.29–1.96)	–	1.22 (1.05–1.42)	–	1.21 (1.07–1.37)
Mother’s perceived baby size^+^					
Average or larger	*Ref*	*Ref*	*Ref*	–	*Ref*
Small or very small	2.73 (2.11–3.52)	1.43 (1.04–1.98)	2.00 (1.63–2.45)	–	1.90 (1.59–2.27)
Mode of delivery^+^					
Non-caesarean	*Ref*	*Ref*	*Ref*	–	–
Caesarean section	1.94 (1.34–2.80)	1.82 (1.14–2.91)	1.84 (1.38–2.46)	–	–

*AHR* Adjusted hazard ratios; 95% CI: 95% Confidence interval

Variables adjusted for: place of residence, wealth index, mother’s (religion, education, age, body mass index, work status and desire for pregnancy), father’s education, child’s (sex, birth place, body size, mode of delivery, delivery assistance, birth order and birth

Interval). Multiple births were excluded from the analysis

Caesarean section is a combination of both elective and emergency caesareans; − variables that were not statistically significant

### Risk factors for post-neonatal mortality (1–11 months)

Post-neonates born to younger mothers (age < 20 and 20–29 years) reported a significantly higher risk of post-neonatal deaths compared to those born to mothers aged between 30 and 39 years (Table 2). Post-neonates born to fathers with no education were more likely to die compared to those born to fathers with secondary or higher education. Other significant factors that affected post-neonatal mortality included delivery by caesarean section, mother’s perceived baby size to be small or very small, and having a fourth or higher birth order with a short or long birth interval ≤ 2 or > 2 years (Table 2).

### Risk factors for infant mortality (0–11 months)

Infants born to younger mothers (< 20 and 20–29 years) were more likely to die before their one-year birthday compared to those born to mothers aged 30–39 years (Table 2). In comparison to female infants, male infants were more likely to die, as were infants perceived to be of small or very small size. Infants born via caesarean section and those who were at fourth or higher birth order with a short birth interval ≤ 2 years had higher risk of mortality compared to those who were at second or third birth order with a birth interval of more than 2 years. Infants whose mothers resided in rural areas were less likely to die compared to those whose mothers lived in urban areas (Table 2).

### Risk factors for child mortality (12–59 months)

Children whose mothers lived in rural areas had a significantly lower risk of deaths compared to those whose mothers resided in urban areas (Table 2). In comparison to children from wealthier households, children from poor or middle households were more likely to die, as were children whose mothers had primary or no educational qualification.

### Risk factors for under-five mortality (0–59 months)

Children under-5 years whose mothers had primary or no education were more likely to die before their fifth birthday compared to those whose mothers had secondary or higher level of education (Table 2). Other significant factors associated with U5M included having a fourth or higher birth order with a short birth interval ≤ 2 years, and mother perception of a small or very small baby size. Children under-5 years born to mothers who resided in rural were less likely to die compared to those whose mothers lived in urban areas.

## Discussion

Over the study period (2004–2016), we found that NMR has remained relatively constant, while PMR and CMR have halved in Tanzania. Similarly, IMR and U5MR have declined over the same period. The improvement in IMR stem from PMR gains, and the reduction in both PMR and CMR led to changes in U5MR, a composite indicator of IMR and CMR. Risk factors for each of the outcome measures varied in our analyses and are discussed in detail below.

The study found that neonates and infants from younger mothers (< 20 years) had a higher risk of death compared to those from older mothers (30–39 years), consistent with evidence from Nigeria [14, 20] and Bangladesh, [27] which indicated that the risk of neonatal, postneonatal and infant mortalities were higher among younger mothers compared to older mothers. Plausible reasons for this findings may be due to the mother’s young age associated with poor health-seeking behaviour [28], and the mother’s experience in optimal infant feeding [29]. However, studies from Tanzania and Swaziland have found no association between maternal age and neonatal mortality [30, 31].

Our analyses revealed that neonates born to mothers who resided in rural areas had a lower risk of neonatal death compared to those living in urban areas, which is similar to findings from Tanzanian reports [12, 32]. However, our finding was inconsistent with a previous study from Nigeria which found that residing in rural area was associated with a higher risk of neonatal mortality [20]. The finding from the present study may reflect poor accessibility and availability of appropriate health facilities and personnel in crowded living conditions in urban slums in many developing countries [33, 34]. In past the two decades, the Government of Tanzania has made significant improvement in providing healthcare access to mothers in rural areas and this may have played a role in our findings in reducing neonatal mortality [10]. Most importantly, improving antenatal care attendance [35], skilled delivery care [36, 37] and postnatal care [35] would potentially have substantial positive impacts on neonatal mortality and U5M in Tanzania. Additionally, addressing cultural and socioeconomic barriers to optimal maternal and newborn care; implementing preventive interventions (e.g., increasing exclusive breastfeeding); and prompt identification and effective case management of sick newborns will help to reduce neonatal mortality in Tanzania [38–41].

Additional factors that were associated with neonatal and infant mortalities included caesarean delivery and perceived small or very small baby size. Reasons for the increased risk of neonatal and infant deaths associated with caesarean delivery in Tanzania include sub-optimal management of caesarean section deliveries, and a lack of awareness and inappropriate use of evidence-based guidelines [42–46]. Similarly, the distribution of inequitable health services and poor referral systems have also been flagged as significant impediments to timely, safe and quality caesarean section deliveries for Tanzanian mothers with resultant poor health outcomes for their infants [47, 48]. Using culturally-appropriate and population-based approaches, Tanzania would do well to improve health and educational messages of women, families and communities associated with strong political commitments to ensure high-quality and comprehensive management of caesarean deliveries with subsequent impact on child survival [49].

Our study suggests that male neonates, infants and U5 children had a higher risk of death compared to their female counterparts, in line with findings from Nigeria which revealed that male infants and under-five children had a higher risk of death [14]. Previous studies have indicated that possible biological and behavioural vulnerabilities may be responsible for this finding, where male neonates and infants were more likely to die compared to females [50, 51]. Furthermore, the analyses showed that children of fourth or higher birth order with short-interval births (≤2 years) were at a higher risk of deaths for neonatal, postneonatal, infant and U5 mortalities. This finding was in line with previous studies which suggested that birth interval and birth order were risk factors for neonatal, postneonatal, infant and U5 mortalities [14, 20, 52]. A reason for this finding might be due to the impact of appropriate family planning as evidence suggest that obstetric complications are higher in women with short birth interval compared to those with long birth interval [52]. Similarly, a possible mechanism for birth order as a risk factor for U5M may be attributable to internal competition for food and other limited household resources, as well as increased demand on the mother [52]. Facility- and community-based interventions to promote family planning would be needed to improve child survival in Tanzania.

The present study also revealed that being a firstborn child was a risk for neonatal and under-five mortality, which may also reflect a mother’s poor access to appropriate healthcare services [28]. Previous small-scale studies from Malawi and The Gambia also reported significant association between primiparity and neonatal mortality [53, 54]. The impact of primiparity on neonatal mortality in sub-Saharan African countries has been attributed to young maternal age, increased risk for infection (such as malaria), preterm deliveries and low birthweight babies among primiparous women [54, 55].

The study indicated that babies who were perceived by the mother to be small or very small in size were more likely to die in their neonatal, post-neonatal, infant or under-5 years’ period compared to those who were perceived to be of average or larger size, consistent with previous studies [14, 20, 56]. This finding may be elucidated by the impact of common aetiological factors (such as prematurity and low birth weight) in those age group and population [56]. We also found that poor or middle household wealth and primary and no educational achievement were associated with child mortality compared to rich households and secondary or higher educational attainment, respectively. Similarly, primary educational achievement was associated with U5M compared to secondary or higher educational achievement. These findings were consistent with previous reports which suggest that lower wealth quintile and no maternal education were associated with child mortality [11, 14, 57]. Empirical evidence has shown that improved maternal education is one of the most important measures to improve not only maternal and child health but also household productivity and socio-emotional interactions of both the mother and her family [58, 59]. The achievement of key SDGs (Goal 1 – end poverty; Goal 4 – ensure quality education and Goal 5 – empower women and girls) will improve health outcomes and socioeconomic positions of Tanzanian women, with positive impact of child survival.

Policy decision-makers, public health experts and other key stakeholders in Tanzania are taking giant steps to ensure that neonatal, post-neonatal, infant, child and U5 mortalities remain on a downward trajectory [60]. For example, in 2016, the Government of the United Republic of Tanzania launched the National Multisectoral Nutrition Action Plan with the aim of improving malnutrition – a major source of childhood mortality in Tanzania. Additionally, the country is also drawing capacities and experiences from the implementation of the Millennium Development Goal (MDG) agenda, where Tanzania met its obligation to the MDG-4 (reduced child mortality), particularly in the context of political will and commitment to good governance and reduction of poverty and disease [10, 61]. Nevertheless, comprehensive and context-specific actions are still needed to improve NMR, as has been with PMR and CMR in Tanzania. These efforts may include considerable investments in health system strengthening (including improving the availability of healthcare workers and infrastructure gaps), education of the girl child and continued political will [62].

The present study has specific limitations. First, we used pooled cross-sectional data across time, where study populations may not be similar. However, we adjusted for period and intra-cluster variability, using the appropriate statistical methodology for a complex survey sample [63]. Second, the assessment of a temporal sequence was not possible as the study used cross-sectional data. Nonetheless, our findings were consistent with previously published studies from sub-Saharan Africa [12, 14, 53, 54, 64]. Third, the study findings may be affected by unmeasured confounding factors such as maternal medical conditions during the pregnancy, the impact of health system and healthcare access on U5M [28] or discrete geographical inequalities [65]. Finally, the study was based on self-reported measures with subsequent potential effect of recall bias on the findings, which may either over- or under-estimate our measure of association between the study factors and outcomes variables.

## Conclusion

The study found that NMR has remained unchanged between 2004 and 2016 in Tanzania, while PMR and CMR have dropped by 50%. Similarly, IMR and U5MR have declined over the same period in Tanzania driven by gains from PMR and a combination of PMR and CMR, respectively. Determinants of neonatal, postneonatal and infant mortalities included caesarean delivery, younger maternal age (< 20 years) and small or very small perceived baby size and fourth or higher birth rank with a short preceding birth interval (≤2 years). Additionally, no maternal education or primary education and poor household wealth were risk factors for child and under-five mortalities.

Despite significant improvements in U5M, unfinished work remains in Tanzania to achieve the recently endorsed global target of ending preventable deaths by 2030. Intervention strategies such as increasing health service contacts for mothers are likely to improve neonatal outcomes. Also, health system investments, the provision of educational messages about birth spacing, empowerment of women and increasing female education could improve child survival in Tanzania.

## Additional file

Additional file 1: **Table S1.** Model for neonatal mortality. **Table S2.** Model for post-neonatal mortality. **Table S3.** Model for infant mortality. **Table S4.** Model for child mortality. **Table S5.** Model for under-5 mortality. **Table S6.** Distribution of under-five mortality by study factors in Tanzania, 2004–2016 (*n* = 1585). (PDF 688 kb)

## Data Availability

The analysis was based on the Tanzania Demographic and Health Survey (DHS) data sets for the years 2004–2005, 2010 and 2015–2016, with some restriction imposed by the DHS program. Approval to use these data was sought from Measure DHS/ICF International, and permission was granted for this use. The data are available online and can be applied for at https://dhsprogram.com/data/available-datasets.cfm. Contact information for data access: The DHS Program Office, ICF, 530 Gaither Road, Suite 500, Rockville, MD 20850. Tel: + 1 301 407–6500; Fax: + 1 301 407–6501; email: info@dhsprogram.com

## Abbreviations

CMR: Child mortality rate
ICF: Inner City Fund
IMR: Infant mortality rate
MDG: Millennium Development Goal
NBS: National Bureau of Statistics
NMR: Neonatal mortality rate
PMR: Postneonatal mortlaity rate
SDG: Sustainable Development Goal
TDHS: Tanzania demographic and health survey
U5MR: Under-five mortality rate
