# Supplementary material for: Determinants of trends in neonatal, post-neonatal, infant, child and under-five mortalities in Tanzania from 2004 to 2016
Source: BMC Public Health. 2019 Sep 9;19:1243. doi: 10.1186/s12889-019-7547-x (PMC6734430; doi:10.1186/s12889-019-7547-x)
Supplement: Supplementary file 1 — Table S1. Model for neonatal mortality. Table S2. Model for post-neonatal mortality. Table S3. Model for infant mortality. Table S4. Model for child mortality. Table S5. Model for under-5 mortality. Table S6. Distribution of under-five mortality by study factors in Tanzania, 2004–2016 (n = 1585). (PDF 688 kb) [file 12889_2019_7547_MOESM1_ESM.pdf]

Supplementary table 1: Model for neonatal mortality

**Variables**	**Stage 1**	**Stage 2**	**Stage 3**	**Stage 4**
Community level factor	HR (95%CI)	HR (95%CI)	HR (95%CI)	HR (95%CI)
**Year of survey⁺**				
2004-2005	*Ref*	*Ref*	*Ref*	*Ref*
2010	0.8292399	0.7891305	0.7836536	0.7907491
2015-2016	0.7781147	0.7099597*	0.755126*	0.7488134*
**Residence type**				
Urban	*Ref*	*Ref*		
Rural	0.5945386*	0.6625102*	0.6256033*	0.6131622*
Socioeconomic level factor				
**Household wealth index**				
Rich		*Ref*		
Middle		1.231725		
Poor		1.275143		
**Mother's education**				
Secondary or higher		*Ref*	*Ref*	*Ref*
Primary		0.9216009	0.8858988	1.018619
No education		0.6229615*	0.6339145*	0.7231685
**Mother's working status**				
Working		*Ref*		
Not working		0.810773		
**Father's education**				
Secondary or higher		*Ref*		
Primary		0.7597502		
No education		0.81459		
Individual level factor				
**Mother's age (Years)**				
30—39			*Ref*	*Ref*
< 20			3.346451*	3.296308*
20—29			0.968163	0.9741225
40—49			0.7684305	0.759587
**Mother's body mass index (MBMI, kg/m2)**				
MBMI > 18.5			*Ref*	
MBMI ≤ 18,5			1.028026	
**Wanted pregnancy at the time**				
Wanted then			*Ref*	
Wanted later			0.8778982	
No more			1.225904	
**Birth rank and birth interval**				
2nd or 3rd child, interval > 2			*Ref*	*Ref*
First child			1.456173*	1.44753*
2nd or 3rd child, interval ≤ 2			1.488027*	1.416315

4th or more child, interval > 2	1.077369	1.081848
4th or more child, interval ≤ 2	1.945828*	1.905949*
**Sex**		
Female	*Ref*	*Ref*
Male	1.614066*	1.590322*
**Mother's perceived baby size at birth**		
Average or larger	*Ref*	*Ref*
Small or very small	2.798389*	2.725938*
Healthcare services		
**Delivery assistance**		
Health professional		*Ref*
Non-health professional		1.189167
**Mode of delivery**		
Non-caesarean		*Ref*
Caesarean section		1.935259*
**Place of delivery**		
Health facility		*Ref*
Home		0.7522289

Notes: $^{+}$; Year of survey was included in all the models irrespective of its significance level; Stage 1- Independent variables (year of survey & community level factor); Stage 2- significant variable(s) in Stage 1 plus socioeconomic variables (maternal education, paternal education, mothers work status & household wealth status); Stage 3 – significant variable(s) in Stage 2 plus individual level factor for maternal and child (mothers age, MBMI, mother's desire for pregnancy, child sex, baby size at birth, & birth order/interval); Stage 4 – significant variables in Stage 3 plus health service factor (delivery assistant, mode of delivery & place of delivery). HR (95%CI): Hazard ratio with corresponding 95% confidence interval

Supplementary table 2: Model for post-neonatal mortality

Variables	Stage 1	Stage 2	Stage 3	Stage 4
Community level factor	HR (95%CI)	HR (95%CI)	HR (95%CI)	HR (95%CI)
**Year of survey⁺**				
2004-2005	*Ref*	*Ref*	*Ref*	*Ref*
2010	0.6827234*	0.6901141*	0.6640418*	0.6586851*
2015-2016	0.4751844*	0.4668802*	0.4590798*	0.454784*
**Residence type**				
Urban	*Ref*			
Rural	1.184483			
Socioeconomic level factor				
**Household wealth index**				
Rich		*Ref*		
Middle		0.9528148		
Poor		0.9844441		
**Mother's education**				
Secondary or higher		*Ref*		
Primary		1.240005		
No education		1.36806		
**Mother's working status**				
Working		*Ref*		
Not working		1.183952		
**Father's education**				
Secondary or higher		*Ref*	*Ref*	*Ref*
Primary		1.092622	1.073352	1.122373
No education		1.46924*	1.436054*	1.556047*
Individual level factor				
**Mother's age (Years)**				
30—39			*Ref*	*Ref*
< 20			6.228211*	6.510216*
20—29			1.816578*	1.963145*
40—49			1.0038	1.028819
**Mother's body mass index (MBMI, kg/m2)**				
MBMI > 18.5			*Ref*	
MBMI ≤ 18,5			0.9182471	
**Wanted pregnancy at the time**				
Wanted then			*Ref*	
Wanted later			0.6615135	
No more			1.324899	
**Birth rank and birth interval**				
2nd or 3rd child, interval > 2			*Ref*	*Ref*
First child			0.8223472	0.8232425
2nd or 3rd child, interval ≤ 2			1.288085	1.124177
4th or more child, interval > 2			1.538788*	1.671436*
4th or more child, interval ≤ 2			1.872942*	1.977023*
**Sex**				

Female	*Ref*	
Male	0.9694235	
**Mother's perceived baby size at birth**		
Average or larger	*Ref*	*Ref*
Small or very small	1.471389*	1.452677*
Healthcare services		
**Delivery assistance**		
Health professional		*Ref*
Non-health professional		1.657962
**Mode of delivery**		
Non-caesarean		*Ref*
Caesarean section		1.829793*
**Place of delivery**		
Health facility		*Ref*
Home		0.9486266

Notes: $^{+}$; Year of survey was included in all the models irrespective of its significance level; Stage 1- Independent variables (year of survey & community level factor); Stage 2- significant variable(s) in Stage 1 plus socioeconomic variables (maternal education, paternal education, mothers work status & household wealth status); Stage 3 – significant variable(s) in Stage 2 plus individual level factor for maternal and child (mothers age, MBMI, mother’s desire for pregnancy, child sex, baby size at birth, & birth order/interval); Stage 4 – significant variables in Stage 3 plus health service factor (delivery assistant, mode of delivery & place of delivery). HR (95%CI): Hazard ratio with corresponding 95% confidence interval

Supplementary table 3: Model for infant mortality

**Variables**	**Stage 1**	**Stage 2**	**Stage 3**	**Stage 4**
Community level factor	HR (95%CI)	HR (95%CI)	HR (95%CI)	HR (95%CI)
**Year of survey**⁺				
2004-2005	*Ref*	*Ref*	*Ref*	*Ref*
2010	0.7508689*	0.7360093*	0.7148601*	0.7077062*
2015-2016	0.6187352*	0.585274*	0.6219872*	0.6055761*
**Residence type**				
Urban	*Ref*	*Ref*	*Ref*	*Ref*
Rural	0.8002898*	0.8779659*	0.7792129*	0.7974976*
Socioeconomic level factor				
**Household wealth index**				
Rich		*Ref*		
Middle		1.036619		
Poor		1.054404		
**Mother's education**				
Secondary or higher		*Ref*		
Primary		1.018095		
No education		0.9006739		
**Mother's working status**				
Working		*Ref*		
Not working		0.9485328		
**Father's education**				
Secondary or higher		*Ref*	*Ref*	
Primary		0.8655531	1.073352	1.122373
No education		1.051592	1.436054*	1.556047*
Individual level factor				
**Mother's age (Years)**				
30—39			*Ref*	*Ref*
< 20			4.24574*	4.466673*
20—29			1.269822	1.340346*
40—49			0.8937569	0.9026748
**Mother's body mass index (MBMI, kg/m2)**				
MBMI > 18.5			*Ref*	
MBMI ≤ 18,5			0.9827271	
**Wanted pregnancy at the time**				
Wanted then			*Ref*	
Wanted later			0.8090744	
No more			1.211723	
**Birth rank and birth interval**				
2nd or 3rd child, interval > 2			*Ref*	*Ref*
First child			1.125677	1.09573
2nd or 3rd child, interval ≤ 2			1.335934*	1.22894
4th or more child, interval > 2			1.216487	1.294069
4th or more child, interval ≤ 2			1.730086*	1.802716*
**Sex**				

Female	*Ref*	*Ref*
Male	1.235023*	1.21983*
**Mother's perceived baby size at birth**		
Average or larger	*Ref*	*Ref*
Small or very small	2.021688*	2.000901*
Healthcare services		
**Delivery assistance**		
Health professional		*Ref*
Non-health professional		1.246857
**Mode of delivery**		
Non-caesarean		*Ref*
Caesarean section		1.861039*
**Place of delivery**		
Health facility		*Ref*
Home		0.8777461

Notes: $^+$; Year of survey was included in all the models irrespective of its significance level; Stage 1- Independent variables (year of survey & community level factor); Stage 2- significant variable(s) in Stage 1 plus socioeconomic variables (maternal education, paternal education, mothers work status & household wealth status); Stage 3 – significant variable(s) in Stage 2 plus individual level factor for maternal and child (mothers age, MBMI, mother’s desire for pregnancy, child sex, baby size at birth, & birth order/interval); Stage 4 – significant variables in Stage 3 plus health service factor (delivery assistant, mode of delivery & place of delivery). HR (95%CI): Hazard ratio with corresponding 95% confidence interval

Supplementary table 4: Model for child mortality

**Variables**	**Stage 1**	**Stage 2**	**Stage 3**	**Stage 4**
Community level factor	HR (95%CI)	HR (95%CI)	HR (95%CI)	HR (95%CI)
**Year of survey**[+]				
2004-2005	*Ref*	*Ref*	*Ref*	*Ref*
2010	0.5767911*	0.5504255*	0.5661412*	0.5739472*
2015-2016	0.4874735*	0.4391464*	0.484458*	0.4982022*
**Residence type**				
Urban	*Ref*	*Ref*	*Ref*	*Ref*
Rural	0.8714645*	0.6216056*	0.5363797*	0.5518565*
Socioeconomic level factor				
**Household wealth index**				
Rich		*Ref*	*Ref*	*Ref*
Middle		1.97749*	2.042766*	2.026421*
Poor		2.075108*	2.170546*	2.182932*
**Mother's education**				
Secondary or higher		*Ref*	*Ref*	*Ref*
Primary		4.224836*	2.43269*	2.472079*
No education		4.697548*	2.57401*	2.678746*
**Mother's working status**				
Working		*Ref*		
Not working		0.9053102		
**Father's education**				
Secondary or higher		*Ref*		*Ref*
Primary		1.313482		1.122373
No education		0.9971735		1.556047*
Individual level factor				
**Mother's age (Years)**				
30—39			*Ref*	*Ref*
< 20			1.647962	4.466673*
20—29			1.077591	1.340346*
40—49			1.093788	0.9026748
**Mother's body mass index (MBMI, kg/m2)**				
MBMI > 18.5			*Ref*	
MBMI ≤ 18,5			1.066664	
**Wanted pregnancy at the time**				
Wanted then			*Ref*	
Wanted later			1.110838	
No more			0.6084769	
**Birth rank and birth interval**				
2nd or 3rd child, interval > 2			*Ref*	*Ref*
First child			1.039231	1.09573
2nd or 3rd child, interval ≤ 2			1.162415	1.22894
4th or more child, interval > 2			1.071771	1.294069
4th or more child, interval ≤ 2			1.194629	1.802716*
**Sex**				

Female	*Ref*	*Ref*
Male	1.132236	1.21983*
**Mother's perceived baby size at birth**		
Average or larger	*Ref*	*Ref*
Small or very small	1.426828	2.000901*
Healthcare services		
**Delivery assistance**		
Health professional		*Ref*
Non-health professional		0.8520181
**Mode of delivery**		
Non-caesarean		*Ref*
Caesarean section		0.2659233
**Place of delivery**		
Health facility		*Ref*
Home		1.193779

Notes: $^{+}$; Year of survey was included in all the models irrespective of its significance level; Stage 1- Independent variables (year of survey & community level factor); Stage 2- significant variable(s) in Stage 1 plus socioeconomic variables (maternal education, paternal education, mothers work status & household wealth status); Stage 3 – significant variable(s) in Stage 2 plus individual level factor for maternal and child (mothers age, MBMI, mother's desire for pregnancy, child sex, baby size at birth, & birth order/interval); Stage 4 – significant variables in Stage 3 plus health service factor (delivery assistant, mode of delivery & place of delivery). HR (95%CI): Hazard ratio with corresponding 95% confidence interval

Supplementary table 5: Model for under-5 mortality

**Variables**	**Stage 1**	**Stage 2**	**Stage 3**	**Stage 4**
Community level factor	HR (95%CI)	HR (95%CI)	HR (95%CI)	HR (95%CI)
**Year of survey⁺**				
2004-2005	*Ref*	*Ref*	*Ref*	*Ref*
2010	0.7066792*	0.6879827*	0.681689*	0.6867292*
2015-2016	0.5808406*	0.5491945*	0.5925562*	0.5988556*
**Residence type**				
Urban	*Ref*	*Ref*	*Ref*	*Ref*
Rural	0.8354777*	0.8012982*	0.7919333*	0.7889419*
Socioeconomic level factor				
**Household wealth index**				
Rich		*Ref*		
Middle		1.21873		
Poor		1.225496		
**Mother's education**				
Secondary or higher		*Ref*	*Ref*	*Ref*
Primary		1.31015*	1.405698*	1.379892*
No education		1.265346	1.340043*	1.320781*
**Mother's working status**				
Working		*Ref*		
Not working		1.008972		
**Father's education**				
Secondary or higher		*Ref*		
Primary		0.9200016		
No education		0.9845557		
Individual level factor				
**Mother's age (Years)**				
30—39			*Ref*	
< 20			1.150051	
20—29			0.9601819	
40—49			1.152517	
**Mother's body mass index (MBMI, kg/m2)**				
MBMI > 18.5			*Ref*	
MBMI ≤ 18,5			0.9719731	
**Wanted pregnancy at the time**				
Wanted then			*Ref*	*Ref*
Wanted later			0.7698705*	0.7779449*
No more			0.862667	0.8932415
**Birth rank and birth interval**				
2nd or 3rd child, interval > 2			*Ref*	*Ref*
First child			1.365543*	1.385969*
2nd or 3rd child, interval ≤ 2			1.453296*	1.427004*
4th or more child, interval > 2			0.9915921	1.041766
4th or more child, interval ≤ 2			1.528529*	1.582744*
**Sex**				

Female	*Ref*	*Ref*
Male	1.210508*	1.212475*
**Mother's perceived baby size at birth**		
Average or larger	*Ref*	*Ref*
Small or very small	1.903953*	1.900566*
Healthcare services		
**Delivery assistance**		
Health professional		*Ref*
Non-health professional		1.187536
**Mode of delivery**		
Non-caesarean		*Ref*
Caesarean section		1.465811
**Place of delivery**		
Health facility		*Ref*
Home		0.9977666

Notes: ⁺; Year of survey was included in all the models irrespective of its significance level; Stage 1- Independent variables (year of survey & community level factor); Stage 2- significant variable(s) in Stage 1 plus socioeconomic variables (maternal education, paternal education, mothers work status & household wealth status); Stage 3 – significant variable(s) in Stage 2 plus individual level factor for maternal and child (mothers age, MBMI, mother's desire for pregnancy, child sex, baby size at birth, & birth order/interval); Stage 4 – significant variables in Stage 3 plus health service factor (delivery assistant, mode of delivery & place of delivery). HR (95%CI): Hazard ratio with corresponding 95% confidence interval

Supplementary table 6: Distribution of under-five mortality by study factors in Tanzania, 2004–2016 (n=1,585)

Variable	Neonatal n (%)	Post-neonatal n (%)	Infant n (%)	Child n (%)	Under–five n (%)
**Year of survey**					
2004-2005	226 (36.6)	257 (45.6)	483 (40.9)	187 (46.4)	670 (42.3)
2010	181 (29.3)	168 (29.8)	349 (29.5)	106 (26.4)	455 (28.7)
2015-2016	211 (34.2)	138 (24.6)	350 (29.6)	110 (27.2)	460 (29.0)
**Residence type**					
Urban	195 (31.5)	104 (18.4)	299 (25.3)	96 (23.9)	395 (24.9)
Rural	424 (68.5)	459 (81.6)	883 (74.7)	307 (76.1)	1190 (75.1)
**Household wealth index**					
Rich	124 (20.1)	77 (13.6)	201 (17.0)	41 (10.1)	242 (15.2)
Middle	249 (40.2)	217 (38.6)	466 (39.4)	172 (42.6)	638 (40.2)
Poor	246 (39.7)	269 (47.8)	515 (43.6)	191 (47.3)	706 (44.5)
**Mother's education**					
Secondary or higher	64 (10.4)	27 (4.9)	91 (7.8)	11 (2.6)	102 (6.5)
Primary	439 (71.0)	375 (66.7)	814 (69.0)	280 (69.2)	1094 (69.0)
No education	115 (18.6)	160 (28.4)	275 (23.3)	114 (28.2)	389 (24.5)
**Mother's working status**					
Not-working	119 (20.4)	64 (11.9)	183 (16.3)	56 (14.6)	239 (15.9)
Working	465 (79.6)	476 (88.1)	941 (83.7)	326 (85.4)	1267 (84.1)
**Mother's age (Years)**					
30-39	207 (33.5)	171 (30.3)	378 (32.0)	132 (32.6)	510 (32.1)
< 20	59 (9.5)	28 (5.0)	87 (7.3)	17 (4.3)	104 (6.6)
20-29	305 (49.2)	305 (54.3)	610 (51.7)	214 (53.0)	824 (52.0)
40-49	48 (7.8)	58 (10.4)	106 (9.0)	41 (10.2)	147 (9.3)
**Mother's body mass index (MBMI, kg/m2)**					
MBMI > 18.5	570 (92.1)	518 (92.0)	1088 (92.1)	370 (91.6)	1458 (92.0)
MBMI ≤ 18.5	45 (7.3)	37 (6.6)	82 (7.0)	32 (7.8)	114 (7.2)
**Wanted pregnancy at the time**					
Wanted then	462 (74.8)	447 (79.4)	909 (77.0)	307 (76.0)	1216 (76.7)
Wanted later	116 (18.7)	75 (13.3)	191 (16.1)	84 (20.7)	275 (17.3)
Unwanted	27 (4.3)	24 (4.3)	51 (4.3)	10 (2.5)	61 (3.8)
**Father's education**					
Secondary or higher	80 (12.9)	38 (6.7)	118 (10.0)	20 (5.0)	138 (8.7)
Primary	378 (61.0)	356 (63.4)	734 (62.1)	296 (73.3)	1030 (65.0)
No education	79 (12.7)	115 (20.4)	194 (16.4)	55 (13.5)	249 (15.7)
**Birth rank and birth interval**					
2 or 3 child, interval > 2	133 (21.5)	142 (25.3)	275 (23.3)	110 (27.2)	385 (42.3)
First child	212 (34.3)	129 (23.0)	341 (28.9)	92 (22.7)	433 (27.3)

2 or 3 child, interval ≤ 2	50 (8.1)	48 (8.5)	98 (8.3)	33 (8.1)	131 (8.2)
4 or more child, interval > 2	151 (24.3)	178 (31.6)	329 (27.8)	129 (32.1)	458 (28.9)
4 or more child, interval ≤ 2	73 (11.8)	66 (11.7)	139 (11.7)	40 (9.9)	179 (11.3)
**Child sex**					
Female	236 (38.1)	287 (51.1)	523 (44.3)	186 (46.1)	709 (44.7)
Male	383 (61.9)	275 (48.9)	658 (55.7)	218 (53.9)	876 (55.3)
**Mother's perceived baby size**					
Average or larger	460 (74.3)	482 (85.7)	942 (79.7)	350 (86.6)	1292 (81.5)
Small or very small	131 (21.2)	61 (10.9)	192 (16.3)	47 (11.5)	239 (15.1)
**Delivery assistance**					
Health professional	209 (33.7)	176 (31.3)	385 (32.6)	127 (31.5)	512 (32.3)
Non-health professional	299 (48.4)	286 (50.8)	585 (49.5)	201 (49.8)	786 (49.6)
**Mode of delivery**					
Non-caesarean	565 (91.4)	532 (94.6)	1097 (92.9)	396 (98.1)	1493 (94.2)
Caesarean section	54 (8.6)	27 (4.8)	81 (6.8)	7 (1.8)	88 (5.5)
**Place of delivery**					
Health facility	146 (37.6)	95 (25.4)	241 (31.6)	73 (26.3)	314 (30.2)
Home	228 (58.7)	265 (70.9)	493 (64.7)	201 (72.4)	694 (66.8)

Notes: n (%), number (and percentage of deaths) across variables; (%), proportions did not sum up to 100% due to missing values; A non-health professional may be a traditional birth attendant who is often a woman, who assists the mother during childbirth and who initially acquired her skills by delivering babies herself or by working with other traditional birth attendants.
